# COVID’s collateral damage on women’s health: How to resume the path of improvement

**DOI:** 10.18332/ejm/122868

**Published:** 2020-06-19

**Authors:** Ramon Escuriet, Anna M. Arribas, Olga C. Vélez

**Affiliations:** 1 Global Health, Gender and Society Research Group, School of Health Sciences Blanquerna, Universitat Ramon Llull, Barcelona, Spain

**Keywords:** women’s health, perinatal care, COVID-19, maternity organization

Since the onset of the current pandemic, there have been important changes in the organization of sexual and reproductive healthcare services in some health systems. These changes have mainly been aimed at meeting the urgent care needs of people affected by the SARS-CoV-2 virus, which is posing a great challenge for healthcare systems.

The current tensions, triggered by the epidemic, have led to organizational changes in a number of healthcare systems that could represent advances in maternal healthcare. These changes are based on earlier experiences in countries that have implemented organizational models in which healthy women have a midwife as their principal health provider. Such models, which have a proven record of producing optimal results, promote an out-of-hospital setting for childbirth care for healthy women, who are given a choice between different options (birth center, homebirth). In short, this is a step forward that is sustained by overwhelming existing evidence. However, in the context of the crisis, the reorganization of the system generally has a negative impact on the allocation of resources for women’s health, including sexual and reproductive health^1^.

In this area, the last few years have produced encouraging changes in terms of the care that women receive in an institutional environment like the hospital. The model of care that has been promoted places women and their families at the center, offering them greater participation in the decision-making process. In addition, interventions that were once practiced routinely have been abandoned, and spaces have been adapted to promote normal childbirth. All these changes, which have been introduced progressively but at a slow pace, are supported by the positive results obtained at the clinical level and by greater satisfaction expressed by women, who report being more satisfied when the care they receive is respectful of their expectations.

Thus, the evidence points to healthy women having better results if they are supported by a midwife^2^ in non-technologized spaces outside the hospital setting. These elements are not in line with the current status quo within most health care systems, but in some cases, they have been slowly introduced into healthcare organizations. Nonetheless, the pandemic situation may have had a negative impact on the health of women overall, putting millions of pregnant mothers and their babies at great risk due to the global reallocation of sexual and reproductive health priorities and services.

The reorientation of health resources means that the different existing organizational models should be reviewed to help inform a new approach, one that prioritizes models that have shown optimal performance. In other words, it is worth considering that, without a change to the organization of some care models, the system will continue to suffer the consequences of suboptimal performance in providing health services, with the result being a sapping of resources that could be used more efficiently elsewhere.

Therefore, it is worth reflecting on the need to break with established routines in the application of certain practices and to reconsider the implementation of others without sufficient evidence. Meanwhile, we should be open to other practices with proven effectiveness^3^. These elements will undoubtedly be important in the coming years, likely to be marked by more limited resources, as it will be necessary to focus more on adaptability and flexibility than on infrastructure and equipment.

However, to speak of changes in the system, one must take into account both the variation in clinical practice between different institutions and the organizational culture of the institutions that provide health services. We can assume that different parallel organizational cultures exist at the same time and place, but that these distinct cultures surely share certain elements. Consequently, we can say that this set of common elements is a structural factor of the health system itself. This structural aspect may explain, on the one hand, the slowness in the integration of certain changes that are presented as innovative but are not aligned with the existing culture, and on the other hand, the speed in the acceptance of the changes that are viewed as aligned with the dominant status quo. It may also account for the possibility of a sudden ‘step backward’ in situations of systemic tension, as is the case during the current epidemic.

Let us discuss this ‘step backward’. Here, a situation of tension has led to the concentration of childbirth care because of the closure of some services, which in turn has prompted an increase in interventionism with the aim of reducing the time of use of the limited space available for obstetric care. There will eventually be time to examine this further and reflect on how health organizations can evaluate and redress the situation.

In the aftermath of the epidemic, and in light of and the changes occurring around the world, it would be interesting to broaden our reflection. We should think more ambitiously about how to improve the capacity for healthcare and broader social responses that offer solutions that strengthen the health system as a whole.

Many factors could influence the coming changes, but we should bear in mind that no single condition will necessarily be decisive and that many things are likely to change.

Thus, in addition to holding a debate on which measures have been shown to be effective, we must also contemplate the need to reorganize services and rethink the implementation of certain practices and organizational models. Some needed changes may, for a number of reasons, have until now met with resistance, despite being called for by existing evidence.

It would be desirable to implement a series of measures aimed at meeting the real needs of the population, in this case women. These could involve:

- *Adaptation of health care*. It is necessary to respond to the individual needs of each family by offering a new model of integrated, person-centred care and by expanding the choice of places to give birth that are made available to women, in line with current scientific evidence. There is increasing demand for care that is respectful of the physiological process of labour, involving practices that also tend to be more cost-effective.
- *Different clinical management strategies*. A collaborative model that efficiently manages maternity care, keeping in mind that approximately 60% of women are healthy but are nonetheless cared for in highly technologized units, which leads to an inefficient use of resources^4^.
- *New professional roles*. Health organizations must encourage the professionals they employ to develop all their competences autonomously and establish an order of preference for their activities. The best results are obtained when professionals do what they have been trained to do. The carrying out of new roles must be closely linked to research and innovation, but institutions themselves must take the lead in implementing these changes. In the case of maternal health, midwives should be the professionals of reference for low-risk women, and they must be able to exercise their profession with full autonomy. In addition, it is important to consider that midwifery care is not interchangeable with nursing care, even in instances where midwives hold nursing qualifications, given that the skills of both professions need to be continually updated to ensure that they are in line with current practice. Redeployment directly leads to shortages of midwives to care for women who continue to become pregnant and give birth^5^.
- *Primary and community care*. The scope of maternity care includes ample space for health promotion and prevention, both key aspects of primary and community care, where there is still room to incorporate innovative models that improve efficiency in the provision of services.
- *Public health*. Motherhood is one of the life events where it is possible to observe the effects of a model based on the control of a planned system over a natural and, in most cases, physiological phenomenon. It is necessary to review this model and rethink the approach, striving for a salutogenic model that helps create and maintain healthy societies.

These five measures could help inform the debate that is expected to take place among health policy decision makers. It will also be important for organized civil society groups, scientific associations and public entities to play key roles in the construction of the kinds of new health policies that are needed to guarantee the future sustainability of the health system.

## CONFLICTS OF INTEREST

The authors have completed and submitted the ICMJE Form for Disclosure of Potential Conflicts of Interest and none was reported.
